# Telemedicine screening of retinal diseases with a handheld portable non-mydriatic fundus camera

**DOI:** 10.1186/s12886-017-0484-5

**Published:** 2017-06-13

**Authors:** Kai Jin, Haitong Lu, Zhaoan Su, Chuming Cheng, Juan Ye, Dahong Qian

**Affiliations:** 1 Department of Ophthalmology, the Second Affiliated Hospital of Zhejiang University, College of Medicine, Hangzhou, 310009 China; 2 Institute of Translational Medicine, Zhejiang University, Hangzhou, 310016 China; 3 Medimaging Integrated Solution Inc., Hsinchu, 30075 Taiwan; 4 School of Biomedical Engineering, Shanghai Jiao Tong University, Shanghai, 200240 China

**Keywords:** Telemedicine, Retina, Imaging

## Abstract

**Background:**

We modified and reconstructed a high image quality portable non-mydriatic fundus camera and compared it with the tabletop fundus camera to evaluate the efficacy of the new camera in detecting retinal diseases.

**Methods:**

We designed and built a novel portable handheld fundus camera with telemedicine system. The image quality of fundus cameras was compared to that of existing commercial tabletop cameras by taking photographs of 364 eyes from the 254 patients. In all 800 fundus images taken by two camera types, 400 images per camera, were graded with the four image clarity classifications.

**Results:**

Using the portable fundus camera, 63% (252/400) images were graded as excellent overall quality, 20.5% (82/400) were good, 11.75% (47/400) were fair, and 4.75% (19/400) were inadequate. Using the tabletop fundus camera, 70.75% (283/400) images were graded as excellent overall quality, 20.4% (51/400) were good, 13.25% (53/400) were fair, and 3.25% (13/400) were inadequate. Common retinal diseases were easily identified from fundus images obtained from the portable fundus camera.

**Conclusion:**

The new type of non-mydriatic portable fundus camera was qualified to have professional quality of fundus images. The revolutionary screening camera provides a foundational platform which can potentially improve the accessibility of retinal screening programmes.

## Background

Retinal disease can lead to vision loss, which is one of the main causes of blindness. Diabetic retinopathy (DR) is a disease that affects up to 80% of diabetics and one of the most common chronic retinal diseases worldwide. In the Beijing Eye Study, prevalence of DR was 2.9 ± 0.3% (95% confidence interval [CI] 2.3–3.5) per individual, in 2011 [1]. An aging society, improvements in medical infrastructure, and lifestyle changes may profoundly increase the DR prevalence in China in the future. Also, age-related macular degeneration (AMD) is the leading cause of blindness in people older than 65 years worldwide. [2] The prevalence of early AMD in Chinese subjects aged ≥50 years was 9.5% (95% CI, 8.2–10.8) and that of late AMD was 1.0% (95% CI, 0.5–1.5). [3] A series of studies have shown that early treatment of retinopathy is effective, to prevent 90% of severe vision loss. Early detection and management of DR, AMD, and other common retinopathies require an effective screening program.

Fundus photography is a basic and effective tool to screen for common retinal diseases. The first fundus camera dates back to the nineteenth century, which offered a 10° retinal field using flash powder and color film. [4] Recently, research progress in electronic illumination control, non-mydriatic imaging systems, and high-resolution digital image capture has enhanced the capabilities of retinal diseases screening. [5] However, one limitation of traditional fundus imaging is its stationary and bulky nature that can be cumbersome to use in hospitalized patients.

Advancements in telemedicine, particularly via portable devices, likely hold the solution to photograph patients who are very ill or very young to sit at a tabletop camera. Moreover, it allows doctors to review photographs in a timely fashion. [6] Lamirel et al. indicated that portable fundus photography could complement ophthalmologic consultations in emergency medicine settings. [7] Martha et al. showed that teleretinal screening by smartphone fundus photography could detect disease in patients who may not have access to ophthalmologic care. [8] However, these portable devices are not easy to handle comparing with tabletop camera and lack of high quality images on the other hand.

However, a newly described technique using portable cameras has offered low-cost screening, especially with personnel shortages and limited photographic equipment. [9, 10] We present a new kind of handheld portable non-mydriatic fundus camera and compare its effectiveness with the tabletop fundus camera. We also show the feasibility of using such a camera in a variety of clinical situations including teleretinal screening.

## Methods

The Medical Ethic Committee of the Second Affiliated Hospital, Zhejiang University School of Medicine approved this study, and it was compliant with Declaration of Helsinki. All participants signed an informed consent before participation in the study.

### General design description of a handheld fundus camera

**Fig. 1:**
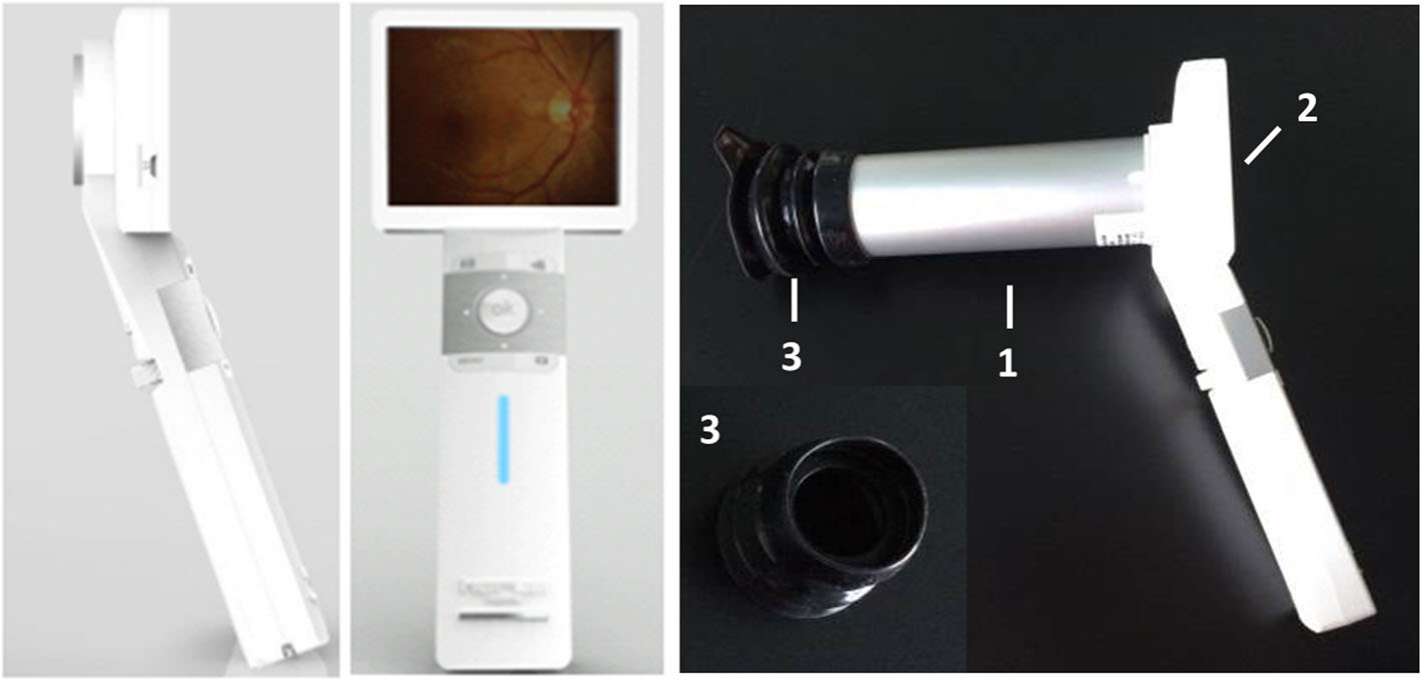
General diagram of the portable fundus camera prototype. The design is separated into two components: (1) an attachment housing optical components and (2) a liquid crystal display (LCD) monitor with a complementary metal-oxide semiconductor (CMOS) sensor. (3) An eyecup can be fixed with on component 1

**Fig. 2:**
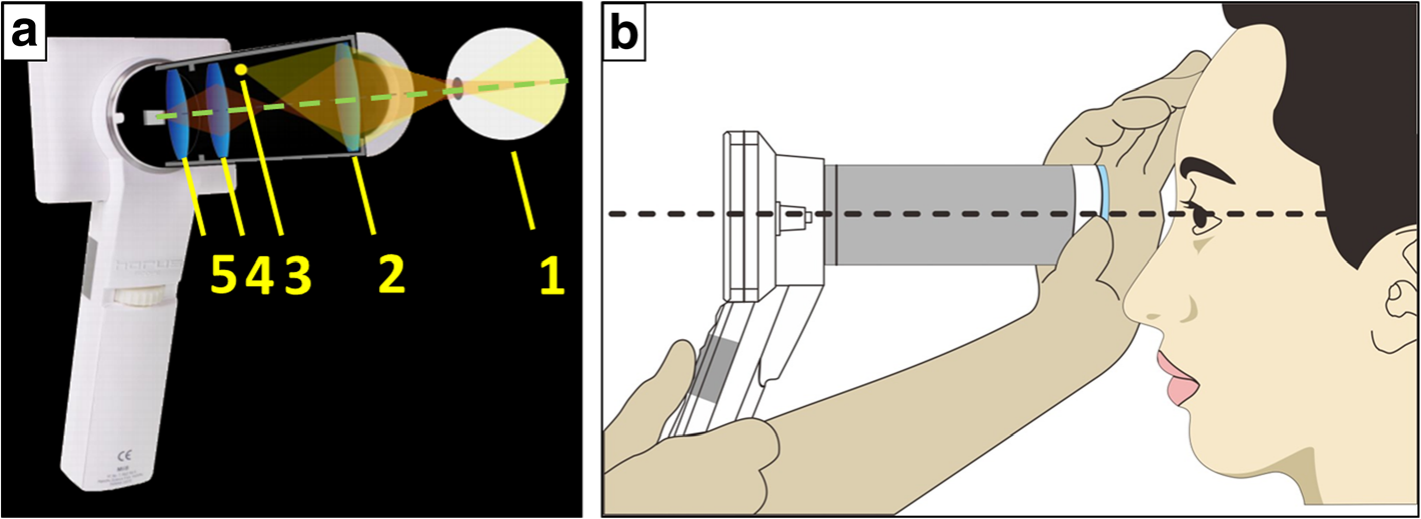
(**a**) Optical configuration of the prototype camera (distances between components are approximated). The components are specified as follows: (1) human eye, (2) front objective lens, (3) condensing lens, (4) visible light emitting diode (LED) unit and xenon flash tube, (5) macro lens. This configuration allows a reflection-free, 60° field–of-view of the fundus in an external housing that can be attached to an LCD monitor with a CMOS sensor that maintains hand-held, point-and-shoot operation. (**b**) Operation of the non-mydriatic portable fundus camera while screening retinal diseases

Ocular fundus camera is based on the principle of indirect ophthalmoscopy. We design a portable optical system based on this imaging principle that is composed of a two-part modular system: an optical attachment that integrates all of the optical components necessary to produce the fundus image (Fig. 1, component 1), and a 3.5-in. full-color TFT-LCD touch panel with a high-definition complementary metal-oxide-semiconductor (CMOS) sensor that can compose, capture, and store the fundus image (Fig. 1, component 2). A detailed schematic diagram of the components within the optical attachment component is shown in Fig. 2a. A front objective lens was positioned at a working distance of 24 mm from the front of the eye. This lens was used to simultaneously relay light rays toward the eye, collect the reflected light, and provide a magnified view of the fundus. All camera components were designed and built by Medimaging Integrated Solution Inc. (MiiS, Taiwan). The final prototype of the portable fundus camera measured approximately 160 × 90 × 200 mm.

### Fundus imaging

The function of the optical attachment was to provide the imaging path (Fig. 1, component 1) to capture an image of the retina. A 20-diopter (D) indirect ophthalmic lens (Fig. 2a, component 2) owing a 60° field of view was used as the front objective lens of the fundus camera. The camera has a number of desirable features including a large 8-megapixel (MP) CMOS sensor, rapid automatic focus and exposure abilities, live view imaging, interchangeable lensing, and built-in image stabilization. The front objective lens, which operates with a working distance of 24 mm, is placed co-axially from the front lens of the consumer. The variability in axial length and refractive error in patients’ eyes are corrected by the auto-focus mechanism of the fundus camera. Retinal illumination is provided by a white light emitting diode (LED) (Fig. 2a, component 3).

### Fundus camera operation

After the optical attachment (Fig. 1, component 1) is attached to the control unit (Fig. 1, component 2) and setup is completed, the user can start taking images. The following steps are to be followed: Choose fixation LED position and ask the patient to look at the fixation LED. Hold the control unit with one hand, and use the other hand to hold the front side of the optical attachment. Maintain the optical attachment at the same height of the eye being examined. To stabilize the optical attachment, the user should rest the optical attachment on the part of the hand between the thumb and index finger, and put the thumb and index finger on the examinee’s forehead, as shown in the Fig. 2b. After finding the optic disc, the image optimization will be done by using the focus adjustment keys of the control unit to adjust the focal distance. The distance from the lens tip to the cornea is 24 mm. Finally, the user presses the OK button to take photographs. The captured image will be shown on the screen and subsequently saved on the SD card.

### Clinical testing

In all, 254 patients (age range, 9–84 years) who were willing to undergo photography by two types of cameras were recruited at the Eye Center of Second Affiliated Hospital of Zhejiang University, China. A handheld non-mydriatic DEC200 fundus camera (MiiS, Taiwan) and a tabletop TRC-NW8 fundus camera (TopCon Medical Systems, Tokyo, Japan) were used to photograph 400 eyes from these 254 patients. The patients were not pharmacologic mydriasis for the portable camera Miis DEC200. And the patients performed imaging with TopCon TRC-NW8 camera 30 min after pharmacologic dilation with tropicamide 1% eyedrops. Images from both the cameras were acquired on the same day. This allowed for direct pathologic identification and comparisons between the two camera types. Patients were recruited over a period of 3 months. The characteristics of two types of fundus cameras were detailed in Table.1. Color fundus photographs were taken by two doctors working in the ophthalmological units. The patients were seated in a dark room (approximately 3 lx, to induce physiologic mydriasis) during the photography for both cameras. The portable fundus camera was fixed with an eyecup (Fig. 1, component 3) on component 1 to achieve physiologic mydriasis and improve the maneuvering stability.

**Table 1:** Characteristics of the fundus cameras

	Miis DEC200	TopCon TRC-NW8
Dimension	16 × 9 × 20 cm	32 × 53 × 57 cm
Weight	0.4 kg	26 kg
Angle of View	60°	60°
Light Source	Infrared & white LED	Halogen lamp & Xe tube
Diopter Compensation	-20 D ~ +20 D	+11 D ~ +33 D
Resolution	8 megapixels	18 megapixels
Price	4500 $	50,000 $

### Fundus photographic quality assessment

A previous study reported that visibility of the macular vessels is a good indicator of image clarity. [11] Therefore, the image quality grading scheme with the four image clarity classifications was used for assessing the quality of fundus images captured by the two types of cameras. One senior retina specialists (Z.S.) and one junior retina specialists (K.J.) assessed and diagnosed with the images independently using a 0.275 mm per pixel monitor at a viewing distance of about 30 cm. The graders were masked to the other grader’s assessment and any patient-related data. Disagreement of 36 cases between the 2 graders was adjudicated by a third retina specialist (J.Y.). In excellent quality, small retinal vessels are clearly visible and sharp around the macula. In good quality, small vessels are clearly visible but not sharp around the macula. In fair quality, vessels are not clearly visible around the macula but are of sufficient clarity to identify third generation branches around the macula. In inadequate quality, third generation branches around the macula cannot be identified. For each image, the senior retina specialist (Z.S.) indicated whether any of the following pathologies were present: DR, AMD, and other retinal diseases. The statistical significance between groups was determined by a two population Student’s *t* test using the Origin software. Statistical significance was set at *p* < 0.05.

### Telemedicine system

**Fig. 3:**
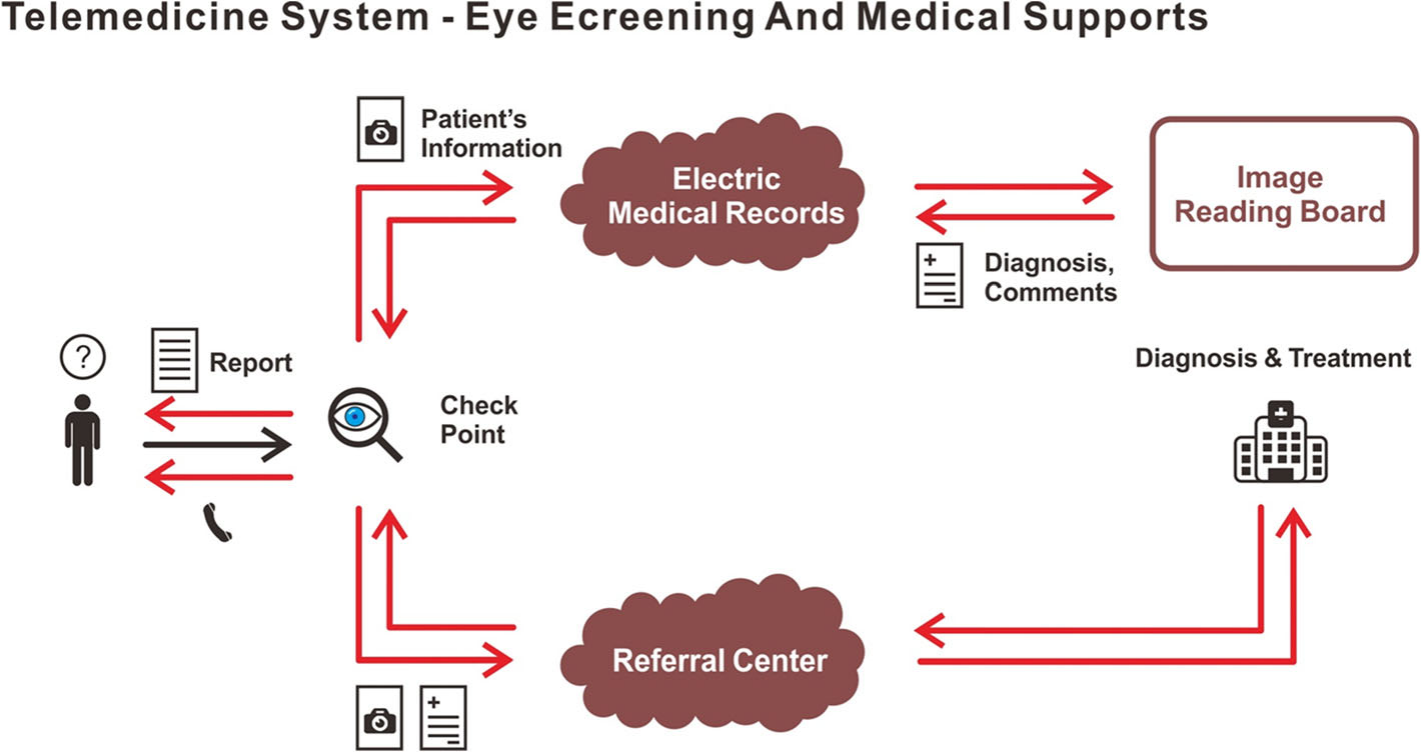
Telemedicine system – retinal diseases screening and medical support

Combined with a 2.4G WiFi transmission system, the fundus photographs from portable fundus camera were uploaded to the electronic medical records (Fig. 3). The images were transmitted from different departments of the Second Affiliated Hospital for diagnoses by the image reading board (Fig. 3) that comprised of retinal specialists. The diagnoses and comments of subjects were transmitted back to the referral center (Fig. 3). After finalization and printing, the reports of the images were sent back to the subjects.

## Results

### Portable fundus camera imaging

The portable fundus camera could be used in a handheld manner with the subject sitting down properly positioned for fundus photography (Fig. 2b). Media opacities, dilated pupil size, and fundus reflection seemed to affect the quality of retinal images. Thus, we photographed each eye twice to ensure satisfactory quality for clinical diagnosis.

The camera was capable of obtaining co-axial 8-MP images of the retina with a 60° field of view. The complete image dimensions were 2560 × 1960 pixels, corresponding to 37 pixels per retinal field degree. This exceeded the minimum image resolution requirement of 6 MP and 30 pixels per degree described by the United Kingdom’s National Health Service for DR screening. [12]

### Photographic quality assessment

**Fig. 4:**
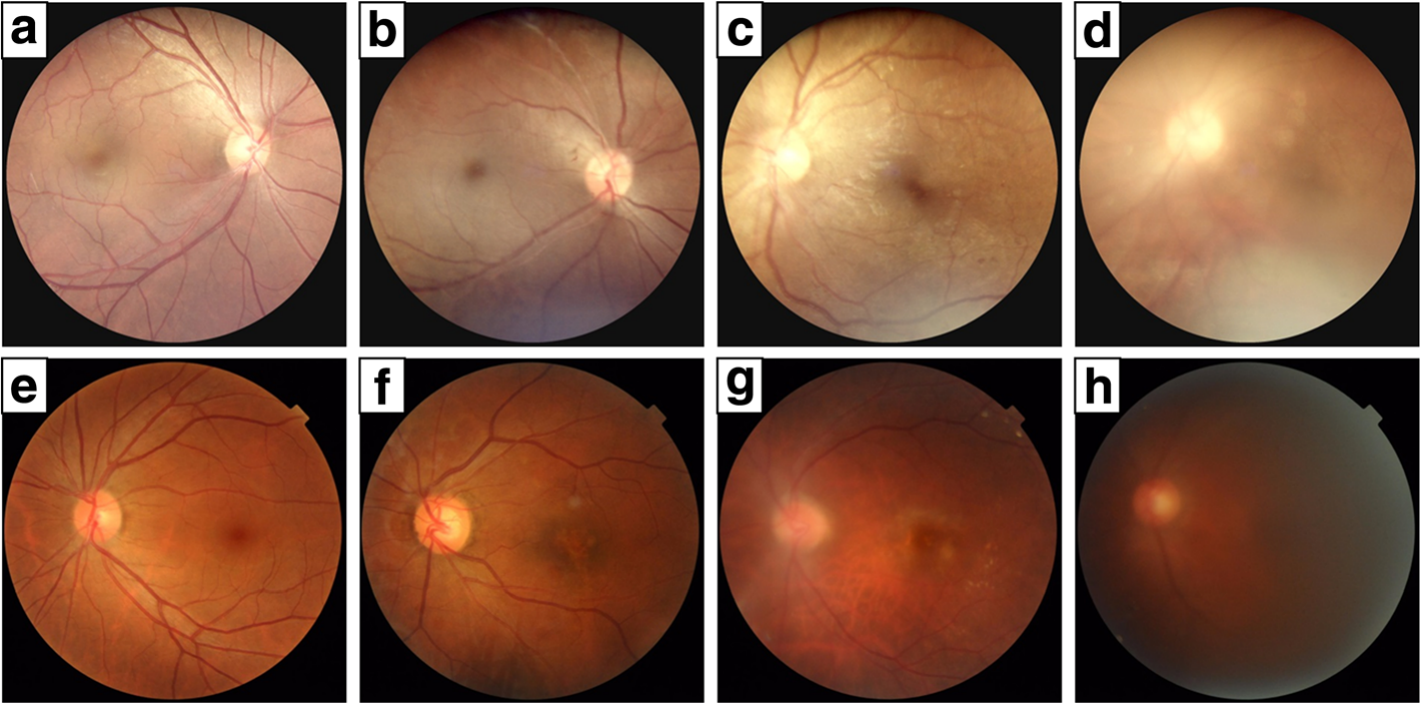
Panels (**a**, **b**, **c**, **d**: Unilateral comparison retinal photographs taken with the DEC200 hand-held portable fundus camera; and Panels (**e**, **f**, **g**, **h)**: Unilateral comparison retinal photographs taken with a commercial TopCon TRC-NW8 camera. The four grading categories of image clarity: (**a**, **e**) excellent; (**b**, **f**) good; (**c**, **g**) fair; and (**d**, **h**) inadequate

We used fundus photographs of 400 eyes obtained by both cameras to compare the cameras’ abilities to accurately detect retinal pathology. In total, 800 photographs were obtained, 400 from each camera. Typical images of photograph clarity classifications by each camera are shown in Fig. 4. Table 2 summarizes the clinician’s gradings of the 800 images. Using the portable fundus camera, 63% (252/400) were graded as excellent overall quality, 20.5% (82/400) were good, 11.75% (47/400) were fair, and 4.75% (19/400) were inadequate. Using the tabletop fundus camera, 70.75% (283/400) were graded as excellent overall quality, 20.4% (51/400) were good, 13.25% (53/400) were fair, and 3.25% (13/400) were inadequate. Although the statistical significance between groups indicated the image quality of tabletop fundus camera was better, the quality of retinal images was comparable with both cameras.

**Table 2:** Photographic quality assessment

	Miis DEC200	TopCon TRC-NW8
Excellent	252 (63%)	283 (70.75%)
Good	82 (20.5%)	51 (12.75%)
Fair	47 (63%)	53 (13.25%)
Inadequate	19 (4.75%)	13 (3.25%)

*P* = 0.015

### Detection of different retinal diseases

**Fig. 5:**
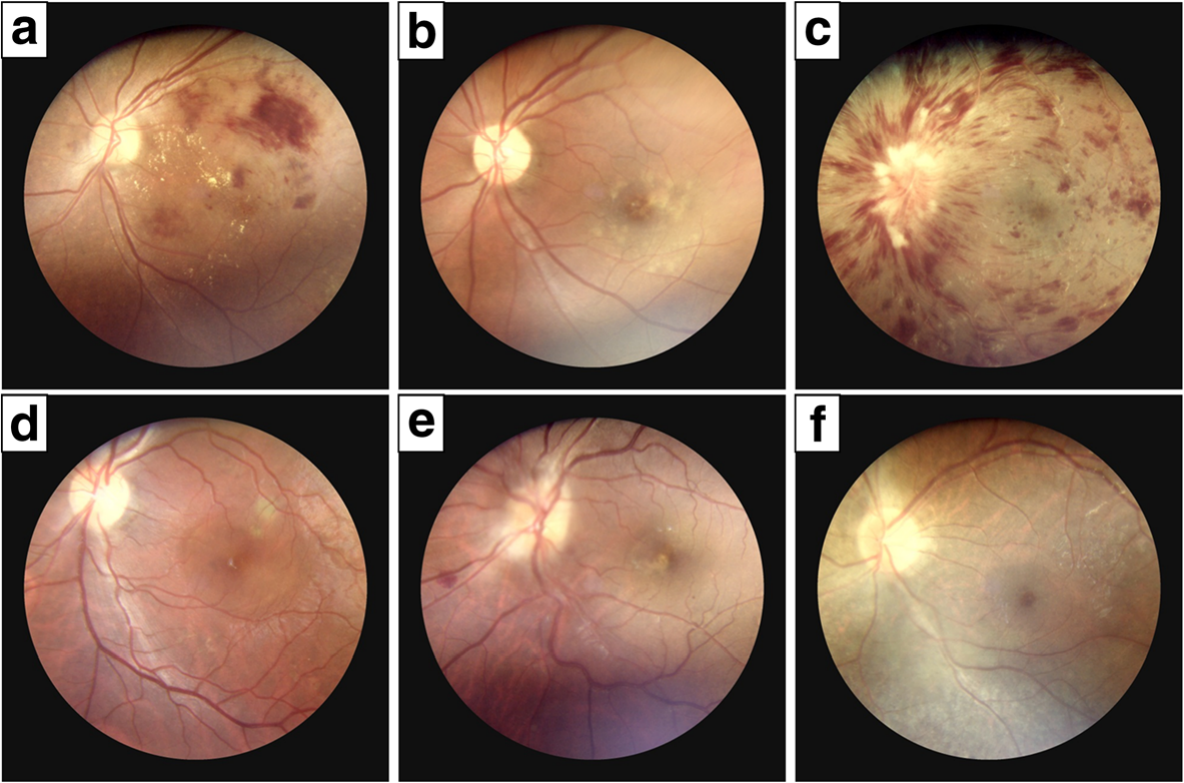
Fundus photographs obtained with the DEC200 portable fundus camera. The unilateral fundus photographs of the study participants show a variety of retinal pathology, including (**a**) diabetic retinopathy with exudate and hemorrhage, (**b**) geographic atrophy with drusen, (**c**) central retinal vein occlusion (CRVO), (**d**) central serous chorioretinopathy, (**e**) papillitis, and (**f**) retinitis pigmentosa (RP)

The portable fundus camera was able to visualize regions of DR with exudate and hemorrhage, geographic atrophy with drusen, CRVO, central serous chorioretinopathy, papillitis, and RP (Fig. 5).

To assess the efficacy between the portable Miis DEC200 fundus camera and the tabletop TopCon TRC-NW8 fundus camera in detecting various retinal diseases, the diagnosis of each image was provided in Table 3. No statistical significance between the two cameras was found, which indicated the comparable efficacy between cameras in detecting different retinal diseases.

**Table 3:** Diagnoses of retinal diseases

	Miis DEC200	TopCon TRC-NW8
DR	129 (32.25%)	131 (32.75%)
AMD	25 (6.25%)	28 (7%)
Normal	142 (3.55%)	139 (34.75%)
Other retinal diseases	85 (21.25%)	89 (22.25%)
Inadequate for diagnoses	19 (4.75%)	13 (3.25%)

*P* > 0.05

## Discussion

We report herein a novel handheld portable retinal camera that can perform high-resolution 60° non-mydriatic retinal imaging, thereby offering specific practical advantages over currently available tabletop fundus cameras. Our portable camera leverages recent advancements in camera technology, including large low-noise image sensors, live view imaging, and image stabilization. The underlying retinal pathology can be immediately identified after image capture by using the camera’s LCD display. We compare Miis DEC200 with three other portable cameras, from the size, field of view, requiring dilating or not and image quality (Table 4). The dimension of the portable fundus camera is approximately 160 × 90 × 200 mm, much smaller than the tabletop TopCon TRC-NW8 fundus camera (320 × 530 × 570 mm) (Table. 1). Tran et al. designed a 50° portable fundus camera to screen for eye disease; the final prototype of their fundus camera measured approximately 245 × 95 × 75 mm. [9]

**Table 4:** Comparison among portable fundus cameras

	Miis DEC200	Tran’s Camera [9]	KOWA Genesis D	Nidek NM-200
Dimension	16 × 9 × 20 cm	245 × 95 × 75 cm	58 × 170 × 81 cm	12 × 23.4 × 26 cm
Field of View	60°	50°	30°(H) / 25°(V)	30°
Pupil Size	≥ 3.5 mm	Not mentioned	≥ 8.0 mm	≥ 4 mm
Requiring Dilating	Unnecessary	Necessary	Necessary	Unnecessary
Resolution	8 megapixels	7.8 megapixels	2 megapixels	1.5 megapixels

Perhaps the need for pharmacologic mydriasis is one of the important limitations of the current fundus cameras. The portable fundus camera used in our study was modified by the underlying optics of the optical attachment to image in the infrared spectrum, and the low intensity illumination won’t cause reflective miosis. Moreover, the time of exposure is so short, thereby enabling non-mydriatic use. Non-mydriatic ocular fundus photography has notable advantages in retinal screening programs and in the emergency department. [13, 14] Without pharmacologic dilation, the fundus image quality of current cameras was significantly affected by the pupil size, especially when the pupils were constricted below 3.5 mm. A recent study by Schwartz et al. indicated that non-mydriatic fundus camera is an effective and feasible screening tool for the early detection of DR. [15] The non-mydriatic color fundus photography and telemedicine succeeded in screening for AMD in their study. Similarly, another study in France showed consistent results for successful AMD screening with a non-mydriatic color fundus photography and telemedicine. [16]

Mobile phone-based retinal cameras are also new type of portable fundus cameras. The iExaminer (Welch Allyn, Skaneateles Falls, New York, USA) can capture retinal images with a mobile phone, but over a limited 25° field of view. Maamari described a novel portable handheld smartphone based retinal camera capable of capturing 55° field fundus images, through a dilated pupil. [10] However, smartphone-based retinal cameras still not got FDA certification.

Portable fundus cameras often remain difficult to use in an ergonomic, handheld fashion. [17] However, our portable fundus camera improved the internal fixation target, and the photographer was able to capture good quality images with little training. Our portable fundus camera photographs had a low rate of ungradable images, and the majority of images were at least of satisfactory quality, which is not very different from the tabletop fundus camera’s low rated images. Yogesan et al. reported that 93% of the fundus images from a handheld fundus camera (Nidek NM-100) were good quality images for diagnosis. [18] However, fixation was difficult.

In teleophthalmology screening of retinal diseases, color images of the retina captured from fundus cameras can be transmitted to a centralized reading center for expert interpretation and diagnosis. [19] However, the previous portable fundus cameras with remote transmission system were all smartphone based. The fundus images taken by our portable fundus camera have been transferred to a cloud server through WiFi connection, although other means of wireless transfer are possible by adding modules such as Bluetooth or a 4G modem. The image reports are then generated by the cloud service and sent back to the subjects. The improved smartphone camera technology offers low-cost eye screening, especially with personnel shortages and limited photographic equipment, even in low- and middle-income countries. [20] Russo et al. reported considerable agreement between smartphone ophthalmoscopy and dilated retinal biomicroscopy in DR grading. [12] In a recent study, the non-mydriatic tabletop fundus camera demonstrated more sensitivity in detecting and grading DR than the smartphone fundus photography. [21] Thus, our non-mydriatic portable fundus camera with remote transmission system may also be promising for use in resource-poor settings.

## Conclusion

In summary, our novel portable non-mydriatic fundus camera with telemedicine system was able to image the fundus with high quality. The revolutionary screening camera provides a foundational platform that can potentially improve the accessibility of retinal screening programs.

## Abbreviations

AMD: Age-related macular degeneration
CI: Confidence interval
CMOS: Complementary metal-oxide-semiconductor
CRVO: Central retinal vein occlusion
DR: Diabetic retinopathy
LED: Light emitting diode
RP: Retinitis pigmentosa
SD: Standard deviation
